# Predicting Potent Compounds Using a Conditional Variational Autoencoder Based upon a New Structure–Potency Fingerprint

**DOI:** 10.3390/biom13020393

**Published:** 2023-02-18

**Authors:** Tiago Janela, Kosuke Takeuchi, Jürgen Bajorath

**Affiliations:** Department of Life Science Informatics and Data Science, B-IT, LIMES Program Unit Chemical Biology and Medicinal Chemistry, Rheinische Friedrich-Wilhelms-Universität, Friedrich-Hirzebruch-Allee 5/6, D-53115 Bonn, Germany

**Keywords:** bioactive compounds, potency prediction, fingerprints, machine learning, conditional variational autoencoder

## Abstract

Prediction of the potency of bioactive compounds generally relies on linear or nonlinear quantitative structure–activity relationship (QSAR) models. Nonlinear models are generated using machine learning methods. We introduce a novel approach for potency prediction that depends on a newly designed molecular fingerprint (FP) representation. This structure–potency fingerprint (SPFP) combines different modules accounting for the structural features of active compounds and their potency values in a single bit string, hence unifying structure and potency representation. This encoding enables the derivation of a conditional variational autoencoder (CVAE) using SPFPs of training compounds and apply the model to predict the SPFP potency module of test compounds using only their structure module as input. The SPFP–CVAE approach correctly predicts the potency values of compounds belonging to different activity classes with an accuracy comparable to support vector regression (SVR), representing the state-of-the-art in the field. In addition, highly potent compounds are predicted with very similar accuracy as SVR and deep neural networks.

## 1. Introduction

Compound potency prediction is a major task in chemoinformatics and computational medicinal chemistry. For potency prediction, both structure- and ligand-based approaches are available. Structure-based methods attempt to predict small molecule (ligand) potency on the basis of experimental (or modeled) three-dimensional (3D) structures of ligand–target complexes. Ideally, such predictions aim to calculate the free energy of binding [1,2], for example, by applying alchemical free energy perturbation methods [2]. These calculations are challenging due to their high computational costs and the need to achieve consistent accuracy across different targets and compound classes [1]. Alternatively, scoring functions of different levels of sophistication are used to approximate ligand binding energies [3,4,5,6].

At the other end of the methodological spectrum reside classical ligand-based approaches for 2D and 3D quantitative structure–activity relationship (QSAR) modeling, which derive linear descriptor-based models for predicting potency values of congeneric compounds (structural analogues) [7,8]. Furthermore, for ligand-based modeling of non-linear SARs and potency prediction, random forest (RF) regression [9] and, in particular, support vector regression (SVR) have become preferred machine learning approaches [10,11]. While SVR typically produces statistically sound prediction models, it also displays a tendency to under-predict the individual most potent compounds because they are often algorithmically classified as outliers [12]. 

The increasing popularity of deep machine learning in pharmaceutical research [13,14,15,16,17] is also influencing structure- and ligand-based potency prediction. One of the attractions of deep learning is the ability to derive new object representations from input data such as molecular graphs, thereby alleviating the need to use pre-conceived molecular descriptors for prediction tasks. Suitable deep neural network (DNN) architectures have been adapted for developing scoring functions [6,7] or deriving ligand–target binding energy models [18,19,20,21]. Despite their apparent success, such models are in part controversially viewed due to the observed strong dependence of their performance on varying training set composition [22,23], resulting from the memorization of training data leading to apparently accurate predictions that do not depend on correctly accounting for ligand–target interactions [22,23,24]. Similar observations have also been made for deep compound classification models with limited generalization ability [25]. In addition to studying ligand–target interactions, DNNs are also intensely investigated for ligand-based molecular property predictions including potency [26,27,28,29]. To these ends, various DNN architectures and learning strategies have been adapted. However, on data sets from medicinal chemistry, which are often limited in size, DNN-based property prediction models often do often exceed—or even meet—the performance of simpler models [29,30]. Hence, for both compound property and potency prediction, no firm conclusion can currently be drawn concerning the potential superiority of DNNs over standard approaches. We have recently shown that k-nearest neighbor (kNN) analysis meets the accuracy of other ML methods in potency prediction [31]. Moreover, randomized predictions typically reproduce experimental potency values within an order of magnitude, which is a direct consequence of potency value distributions in compound activity classes commonly used for benchmarking [31]. Hence, the best ML models and random predictions are only distinguished by a small margin of maximally one order of magnitude, representing a general limitation associated with the benchmarking of potency prediction methods. This needs to be taken into consideration when evaluating these methods, calling for the inclusion of simple controls such as kNN. 

In this work, we introduce a novel concept for compound potency prediction that combines a special fingerprint (FP), termed structure–potency FP (SPFP), with a deep learning approach. FPs accounting for chemical structure and topology are a mainstay for chemical similarity searching [32,33]. SPFP is the first FP representation specifically designed to combine compound structure and potency information in a modular format. Using SPFP, a conditional variational autoencoder (CVAE) [34,35] is trained to predict potency from chemical structure using the structural module of test compounds as input. Given the uniform structure–potency bit string encoding, SPFP–CVAE models do not depend on class labels or associated variables for learning. 

## 2. Materials and Methods 

### 2.1. Compound Activity Classes

Bioactive compounds were extracted from ChEMBL (version 28) [36]. The compounds with a reported direct target interaction (target confidence score: 9) and a numerically specified potency (pIC50) value (standard relation: “=”) were initially retrieved. Then, the compounds with a molecular weight less than 1000 Da and potency values falling into the pIC50 range from 5 to 11 were selected. All the compounds with interactions labeled as “inactive”, “not active”, “inconclusive”, “potential transcription error”, or “pan assay interference compounds” (PAINS) [37] were discarded. Furthermore, the PAINS filter from RDKit, a filter based on liability rules from medicinal chemistry [38], and the aggregation advisor [39] were applied to remove compounds with potential assay interference characteristics. On the basis of these criteria, 132,175 unique compounds were obtained with activity against 1315 human targets. The qualifying compounds were organized into target-based activity classes (pharmaceutical anti-targets were omitted). A set of 10 activity classes was randomly selected from the large pool, comprising 18,231 unique compounds (Table 1) and used for activity class-based model building, hyper-parameter optimization, and model evaluation. 

### 2.2. Model Building and Evaluation

For each activity class, training and test sets were randomly assembled to yield a constant 90:10 compound partition. Across all models, the predictive performance was evaluated over 10 independent trials using different performance measures. For 80:20 compound data partitions used as a control, nearly identical results were obtained.

#### 2.2.1. Conditional Variational Autoencoder

CVAE [40] is an adaptation of the variational autoencoder (VAE) [41], a supervised deep learning algorithm for generative modeling that constructs a conditioned data representation into a continuous latent variable (z). The probabilistic encoder q(z|X, c) (recognition network) uses a condition vector (c) to map the input data to a Gaussian distribution, p(z|c)∼N(0, I) (prior network) into the latent space. The decoder p(X|z, c) then reconstructs data samples from the conditioned latent space to obtain the original input representation (dimensionality). The encoder and decoder are trained with the objective of optimizing the evidence lower bound (ELBO) of the input data [42,43]. During training, the conditioned encoder learns to approximate a latent variable distribution by minimizing the Kullback–Leibler (KL) divergence [44] between data distributions in the original and latent space. The decoder is trained to minimize the reconstruction error of the data representation.

The CVAE encoder and decoder networks consisted of three hidden layers, with 512, 256, and 128 neurons, respectively. For hyper-parameter optimization, a grid search protocol was applied to determine the number of neurons for the latent layer (16, 32, and 64). Different learning rates (0.1, 0.01, and 0.001), dropout rates (0 and 0.5), and batch sizes (16, 32, and 64) were evaluated. Network training was performed with Adam [45] optimizer and the hyperbolic tangent (tanh) was used as the activation function. The parameters β (1 and 2) and σ (0.01, 0.1, and 1) were tested. The learning rate was steadily reduced, during training, to improve learning, and the models were run for a maximum of 150 epochs or until convergence was reached with the early stopping option to avoid network overfitting. The CVAE cost function was computed as the mean of the reconstruction (binary cross-entropy) loss and KL divergence loss. 

#### 2.2.2. Support Vector Regression

The support vector regression (SVR) is a variant of the supervised support vector machine algorithm that derives an ε-insensitive tube based on the training data for the prediction of numerical values, with the maximum permitted error provided by the width of the ε tube [10,11].

For SVR, the cost hyper-parameter C was optimized by testing (0.001, 0.005, 0.01, 0.05, 0.1, 0.5, 1, 10, 100, and 10000) values. The SVR models were built using the Tanimoto kernel [46].

#### 2.2.3. Random Forest Regression

Random forest regression (RFR) is a machine-learning algorithm based on an ensemble of decision trees. During model training, each tree is created by splitting the respective node and bootstrapping aggregation is used to randomly select the training instances. The mean value across all decision trees is used to determine the final prediction [47].

In RFR parameter optimization, the number of decision trees (50, 100, and 200), the minimal number of samples for a split (2, 3, 5, and 10), and the minimum number of leaf-node samples (1, 2, 5, and 10) were used as the search parameter space. 

#### 2.2.4. Deep Neural Network

DNN is a deep learning method capable of mathematically modeling data using a non-linear activation function through the neurons of the network’s fully connected layers. The network learning process consists of interactively determining the difference between the observed and predicted values, using a stochastic gradient descent algorithm to minimize the loss function until it converges to a specific minimum value [48,49].

The DNN models were trained using several network architectures by varying the different numbers of hidden layers (2 and 3) with hyperbolic tangent (tanh) activation, and the network neurons (100–500). Grid searches were performed for different batch sizes (16 and 64), dropout (0 and 0.5), and learning rates (0.1, 0.01, and 0.001). The networks were trained using an Adam optimizer for a maximum of 200 epochs with early termination.

#### 2.2.5. k-Nearest Neighbor Ranking 

kNN is a supervised learning method that ranks the training compounds based on increasing the fingerprint similarity (shortest distance). For the final prediction, the k-top training compounds potency value is accessed (e.g., 1-NN—potency value, and 3-NN—average potency) and assigned to the test compound [50]. For kNN optimization, the optimal k values were evaluated with 1, 3, and 5 top-rated compounds. 

#### 2.2.6. Mean Regression

The mean regressor (MR) approach is based on assigning the mean potency value of the training set to each compound present in the test set. This method was used as a control calculation to generate the random predictions.

#### 2.2.7. Random Predictions

A y-randomization control was performed by the random reassignment of potency values across the compounds from each activity class (random shuffling) [51]. 

#### 2.2.8. Hyperparamters and Implementation 

The kNN, SVR, RF, and SPFP–CVAE model hyperparameters were optimized using an internal five-fold cross-validation, whereas the DNN parameter optimization was performed with an internal 90:10 training–validation split. The SVR, RFR, kNN, and MR models were generated using scikit-learn [52]. The CVAE and DNN models were implemented with Keras [53] and Tensorflow [54].

#### 2.2.9. Evaluation Metrics

The performances of all the models were evaluated by calculating the mean absolute error (MAE) and root mean squared error (RMSE) for predicted and experimental test compound potency values using scikit-learn.

(1) $$MAE\left(y,\hat{y}\right)=\frac{1}{n}\sum_{i=1}^{n}\left|y_{i}-{\hat{y}}_{i}\right|$$

(2) $$RMSE\left(y,\hat{y}\right)=\sqrt{\sum_{i=1}^{n}\frac{{\left(y_{i}-{\hat{y}}_{i}\right)}^{2}}{n}}$$

where n is the number of compounds, and y and ŷ are the experimental and predicted potency values, respectively. 

An assessment of the statistical significance was performed for the value distributions from predictions based on MAE and RMSE values using the nonparametric Wilcoxon test [55]. The null hypothesis was either rejected or accepted, by setting alpha to 0.05 and comparing it to the respective *p*-value (*p* ≤ 0.05).

### 2.3. Molecular Representation

The compounds were represented using a folded version of the extended connectivity fingerprint with bond diameter 4 (ECFP4) [56], which is a generally preferred topological descriptor for many chemoinformatics applications, consisting of layered atom environments, consisting of 2048 bits. The ECFP4 fingerprint was generated using RDKit [57].

The scripts for the reported calculations and the curated activity classes are available from the authors upon request. 

## 3. Results and Discussion

### 3.1. Concept of Potency Prediction Based on Fingerprint-Based Potency Encoding

The introduction of a structure–potency fingerprint (SPFP) provided the basis for a new approach in potency prediction. The underlying idea was to unify structural and potency encodings in a modular fingerprint representation of a constant format such that the potency module representing a numerical value could be predicted from the structural module of test compounds using deep learning. This unified and intuitive modular encoding of compound structure and potency enabled the derivation of a chemical language model such as a CVAE using SPFPs of training compounds to predict the potency module of test compounds using only their structure module as input. 

**Figure 1:**
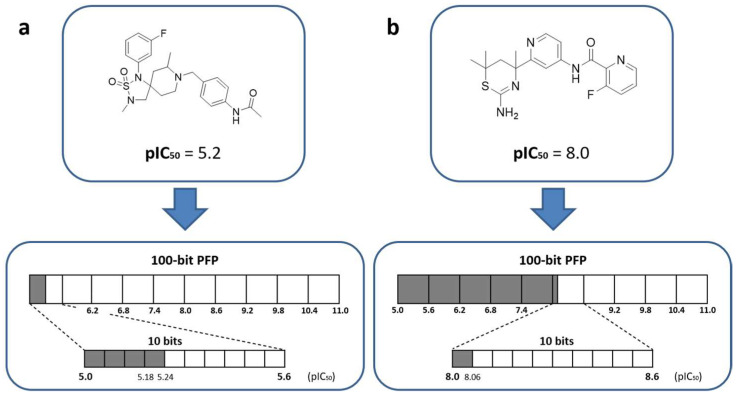
Cumulative potency encoding. (**a**,**b**) illustrate how logarithmic potency values of different magnitude are encoded in the potency module of SPFP.

**Figure 2:**
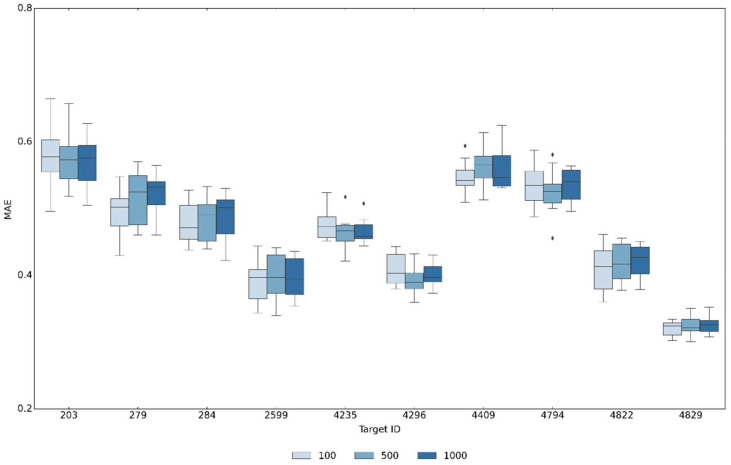
Prediction accuracy for SPSF with differently sized potency modules. Boxplots report mean absolute error (MAE) values of SPFP–CVAE models using alternative SPFP versions with potency modules comprising 100, 500, or 1000 bits evaluated across all activity classes according to Table 1. In boxplots, the upper and lower whiskers indicate maximum and minimum values, the boundaries of the box represent the upper and lower quartiles, values classified as statistical outliers are shown as diamonds, and the median value is indicated by a horizontal line.

An extended connectivity fingerprint with a constant size of 2048 bits represented the structure module of SPFP that was combined with a newly designed potency module for representing compound potency values. We defined two principal requirements for the potency module. Hence, it was required to, first, represent the biologically relevant large (negative decadic logarithmic) potency range from 5 to 11 and, second, encode the potency values at a meaningful resolution such that accurate predictions could in principle be obtained. Therefore, alternative single value, value range, and cumulative coding schemes suitable for bit string representations were initially investigated and cumulative value range encoding was found to be the most robust approach (that is, yielding the most stable predictions across independent trials). Accordingly, contiguous segments of increasing numbers of bits were used to represent increasingly potent compounds populating the entire logarithmic potency range from 5 to 11. For example, Figure 1a,b show how a potency value of 5.2 and 8.0 was encoded by setting on the first four and 51 bits in the potency module, respectively. To meet the second requirement stated above, we set the size of the potency module to a minimum of 100-bit positions such that each individual bit position accounted for 0.06 log units via cumulative potency encoding. Accordingly, the resolution of the potency predictions was intrinsically limited to 6% of a log unit. This level was deemed acceptable for the approach because it fell within the typical range of experimental accuracy limitations. Smaller bit numbers for the potency module would lead to larger resolution limits while larger numbers would further increase the resolution. Therefore, we also tested larger versions of the potency module using the SPFP–CVAE models comprising 500 and 1000 bits, as reported in Figure 2. These control calculations produced very similar results to those obtained for the 100-bit potency module, hence showing that the prediction accuracy could not be further increased by decreasing the resolution limit of the potency encoding and supporting the choice of 100-bit positions for the potency module. Furthermore, for potency predictions using CVAE sampling, a bit module with a constant format and meaningful size was required to assess the predictions in a meaningful way (see below). 

### 3.2. Learning and Prediction Strategy

**Figure 3:**
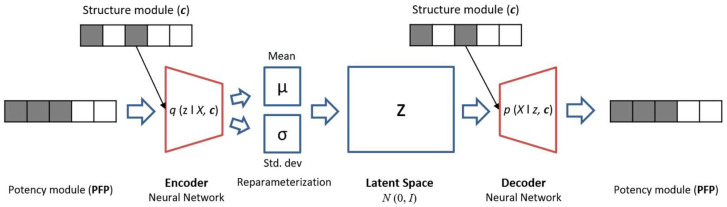
Architecture of the conditional variational autoencoder. The encoder network transforms the potency module (PFP), conditioned by the structure module (c), into a distribution of latent variables (z). The decoder samples a conditioned latent vector from a Gaussian distribution and reconstructs the potency module.

The CVAE model architecture used here consists of an encoder, latent space layer, and decoder, as illustrated in Figure 3. 

**Figure 4:**
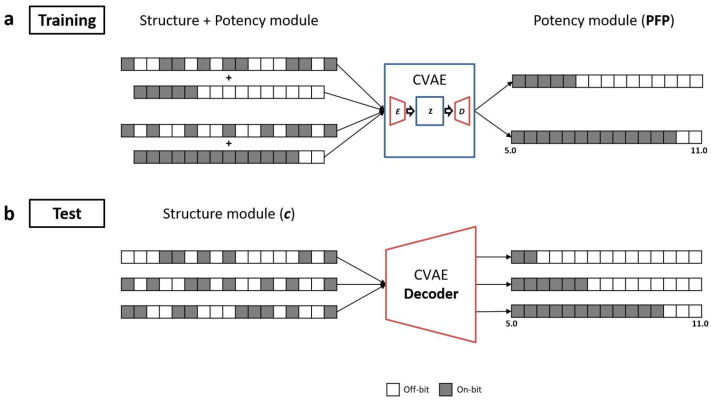
Conditional variational autoencoder modeling. In (**a**,**b**), the CVAE training and prediction strategies are illustrated, as discussed in the text.

For each compound activity class, a CVAE model was trained to reproduce the complete bit patterns of the potency module, conditioned by the structure module, as illustrated in Figure 4a. Each CVAE model was then used to predict the bit settings of the potency module (PFP). Therefore, the potency values of the test compound were predicted by submitting the structure module (c) to the CVAE decoder to generate the corresponding potency module, as illustrated in Figure 4b. Since the CVAE predictions depended on the sampling of potency modules in latent space, the evaluation criteria for potency module variants were defined. Accordingly, for a given test compound, a sampled potency module was classified as valid if it contained a contiguous bit string in which all bits were set on. If this criterion was met, the predicted potency value was assigned to the center of the respective potency interval (e.g., 5.03 for the [5.0–5.06] interval), resulting in a constant standard deviation of ±0.03 log units for all predictions. By contrast, if the output bits were not contiguous, that is, if they were not consistent with the cumulative encoding of the potency module, the prediction was classified as invalid and the sampling was continued until a valid prediction was obtained, given a maximal number of permitted sampling steps. 

### 3.3. Potency Predictions

**Figure 5:**
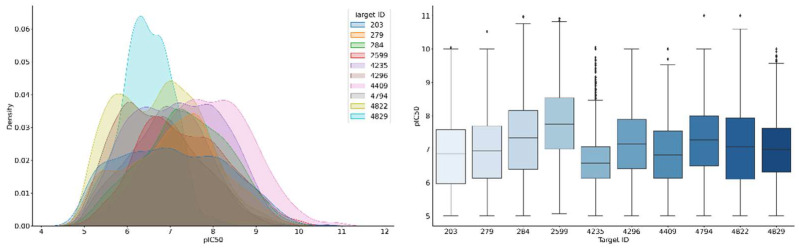
Potency value distribution of activity classes. Kernel density estimation plots (**left**) and boxplots (**right**) compare the potency values distributions of the 10 activity classes. Coloring of boxplots is arbitrary. The horizontal line indicates the median of the value distribution and diamond symbols represent statistical outliers.

For 10 randomly selected compound activity classes, different ML models were generated. Figure 5 shows that the compound potency value distributions of the activity classes were overlapping yet distinct, mostly yielding median potency values in the high nanomolar range. The comparison also shows that logarithmic potency values below 5 (approaching experimental accuracy limitations) and above 10 (sub-nanomolar potency) were generally sparse. 

**Figure 6:**
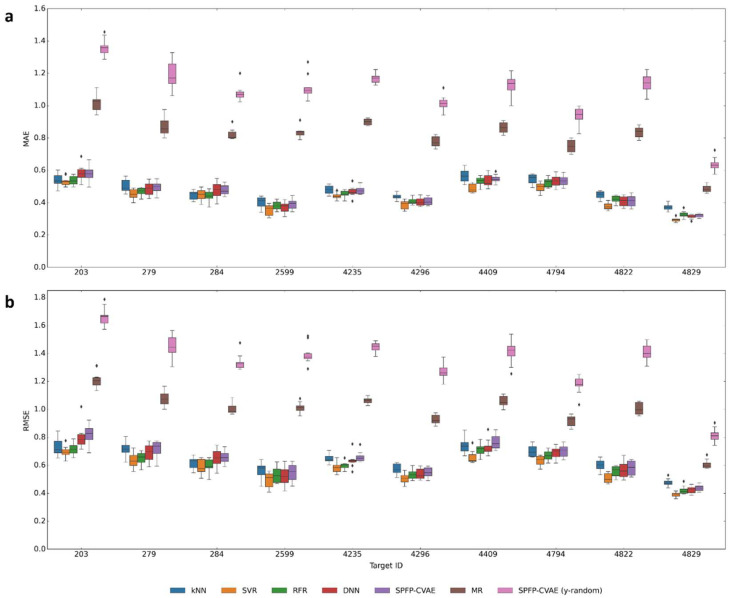
Prediction accuracy. Boxplots report the (**a**) MAE and (**b**) RMSE values for potency predictions using different ML models across all activity classes.

Activity class-dependent potency prediction models were then generated for SPFP–CVAE, k-nearest neighbor (kNN) analysis, SVR, RFR, and DNN. These ML approaches currently represent the state of the art in compound potency prediction [31]. In addition, a mean regressor (MR) was applied as a control, which simply assigned the mean potency value of an activity class to all test compounds. The results are reported in Figure 6. 

Overall, similar prediction accuracy was observed for the different ML models, regardless of their complexity, mostly with median MAE and RMSE of ~0.4–0.5 and ~0.6–0.7, respectively. As observed before [31], simple kNN-based potency assignments approached or exceeded the prediction accuracy of ML models and there was no advantage of deep learning approaches over other ML methods. Moreover, the MR yielded median MAE and RMSE values of ~0.8–0.9 and ~1.0–1.1, respectively. The performance of randomized SPFP–CVAE models was only slightly worse than MR, mostly with a median MAE value of ~1.0–1.2, owing to the dominance of compounds with potency values between 6 and 8 across all activity classes, as reported in Figure 5. These artificial predictions using MR or randomized models reproduced experimental values within about one order of magnitude, providing a limit for prediction accuracy, while most accurate ML models typically achieved mean MAE value of ~0.4. Hence, there was only a relatively small margin between best and artificial predictions, defining a window of less than one order of magnitude in which model performance must be evaluated [31]. In the previous study, equally curated versions of three activity classes (279, 284, 4822) from a different ChEMBL release were investigated using ML methods with different calculation protocols, yielding prediction accuracies very similar to the values reported herein [31]. 

**Figure 7:**
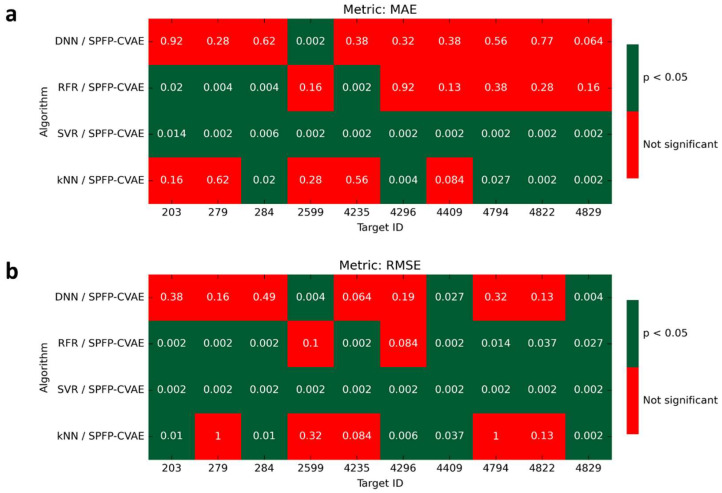
Statistical significance assessment. Statistical significant (Wilcoxon signed-rank) tests based on (**a**) MAE and (**b**) RMSE values were carried out for the performance differences observed between SPFP–CVAE and all other ML models (kNN, SVR, RFR, and DNN). Red cells indicate *p*-values above α = 0.05 (no statistical significance) and green cells *p*-values below α = 0.05 (statistical significance).

Many of the small performance differences observed in Figure 6 were not statistically significant, as reported in Figure 7, while differences between SPFP–CVAE, SVR, and RFR were statistically significant for about half of the activity classes. However, the prediction accuracy of these three approaches was very similar, which was also reflected by the respective *p*-values. Overall, SVR was the preferred approach, but only by a very small margin compared to SPFP–CVAE and other ML methods. For example, the differences in the median MAE between SPFP–CVAE and SVR ranged max. ~0.01–0.02, depending on the activity class, which was marginal at most and would be considered irrelevant for all practical purposes. 

### 3.4. Predicting Highly Potent Compounds

**Figure 8:**
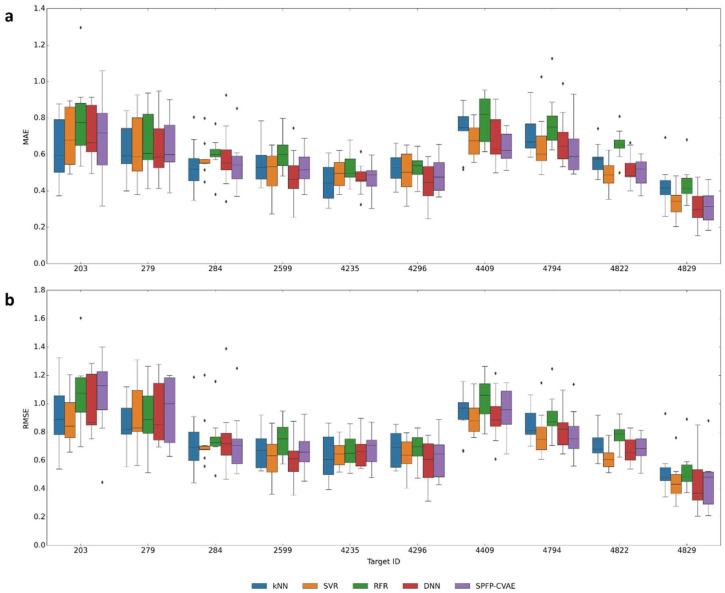
Prediction accuracy for the most potent test compounds. Boxplots report the median MAE (**a**) and RMSE (**b**) values for the 10% most potent test compounds from all classes and different ML models including kNN.

We then investigated the ability of the different ML methods to predict the 10% most potent compounds in a test set (typically amounting to ~15–20 compounds) using models derived based on the original sets. The results for models derived from original training sets are shown in Figure 8. Due to the small test sample size of these predictions, the MAE and RMSE value distributions were broader than for the global predictions reported in Figure 6. 

**Figure 9:**
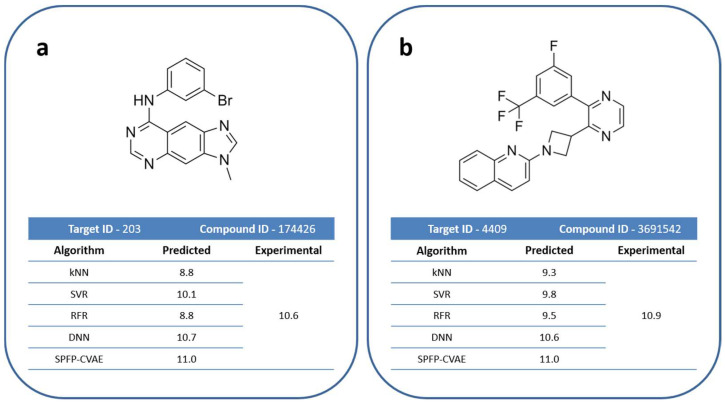
Highly potent compounds. (**a**,**b**) illustrate predictions for two exemplary highly potent test compounds using different methods.

Compared to the global predictions, the median MAE and RMSE value for most potent compounds increased to ~0.6 and ~0.8 or greater, respectively, for about half of the activity classes while the prediction accuracy remained similar to before for the remaining classes. However, the performance of the different ML methods including kNN continued to be comparable (MR was omitted here because of the naturally large deviations for the small number of the most potent compounds). Overall, SVR, SPFP–CVAE, and DNN yielded best predictions with only small (and activity class-dependent) differences between these methods. The predictions for the exemplary compounds are shown in Figure 9. 

## 4. Conclusions

Compound potency prediction is an important task in chemoinformatics and medicinal chemistry. For structure- and ligand-based predictions, different methods have been introduced. QSAR techniques including non-linear modeling using machine learning continue to play an important role. Herein, we have introduced a new methodological concept for compound potency prediction that depends on the newly designed SPFP format for structure–potency encoding and CVAE learning. The SPFP–CVAE concept was devised to enable the prediction of bit settings in SPFP potency modules from input structure modules, without learning correlations between structural representations and potency values used as a dependent variable. In activity class-dependent predictions, the SPFP–CVAE approach essentially met SVR performance, representing the current state of the art in the field. Given the general limitations associated with the potency predictions in benchmark settings, we consider the prediction of most potent compounds a particularly meaningful exercise. In this case, SVR, SPFP–CVAE, and DNN achieved comparable accuracy. Taken together, our results indicate that the SPFP–CVAE concept introduced herein provides a new methodological framework for compound potency prediction that can be further explored in various ways. Importantly, FP-based structure–potency encoding, as introduced herein, can be easily modified for different applications, providing a versatile input format for ML.

## Figures and Tables

**Table 1:** Activity classes. Ten activity classes used for deriving and evaluating activity class-based prediction models are reported.

Target Name	Target ID	# Compounds
Beta-secretase 1	4822	2270
11-beta-hydroxysteroid dehydrogenase 1	4235	2232
Phosphodiesterase 10A	4409	2109
Acetyl-CoA carboxylase 2	4829	1811
Dipeptidyl peptidase IV	284	1709
Sodium channel protein type IX alpha subunit	4296	1703
Tyrosine-protein kinase SYK	2599	1616
Vascular endothelial growth factor receptor 2	279	1614
Epidermal growth factor receptor erbB1	203	1606
Vanilloid receptor	4794	1562

## Data Availability

Compound data sets are publicly available.
